# Cluster Beam Study of (MgSiO_3_)^+^-Based Monomeric Silicate Species and Their Interaction with Oxygen: Implications for Interstellar Astrochemistry

**DOI:** 10.1021/acsearthspacechem.2c00186

**Published:** 2022-10-06

**Authors:** Joan Mariñoso Guiu, Bianca-Andreea Ghejan, Thorsten M. Bernhardt, Joost M. Bakker, Sandra M. Lang, Stefan T. Bromley

**Affiliations:** † Departament de Ciència de Materials i Química Física & Institut de Química Teòrica i Computacional (IQTCUB), Universitat de Barcelona, c/ Martí i Franquès 1-11, 08028 Barcelona, Spain; ‡ Institute of Surface Chemistry and Catalysis, Ulm University, Albert-Einstein-Allee 47, 89069 Ulm, Germany; § Radboud University, Institute for Molecules and Materials, FELIX Laboratory, 6525 ED Nijmegen, The Netherlands; ∥ Institució Catalana de Recerca i Estudis Avançats (ICREA), Passeig Lluis Companys 23, 08010 Barcelona, Spain

**Keywords:** magnesium silicates, interstellar dust, pyroxene monomer, gas phase clusters, infrared spectroscopy, DFT calculations, oxygen depletion, cosmic dust formation

## Abstract

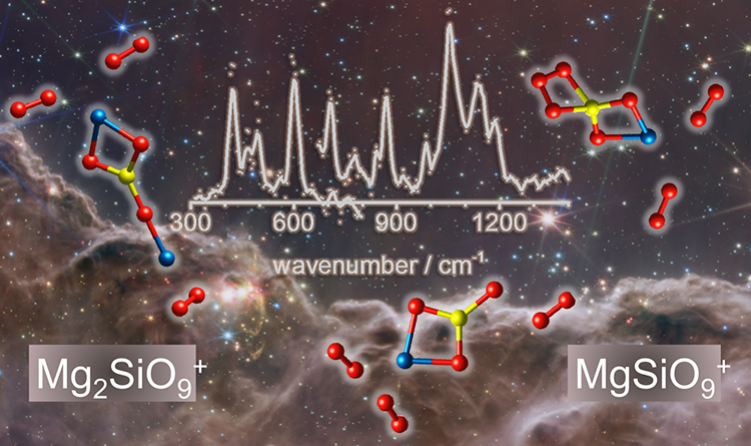

Silicates are ubiquitously found as small dust grains throughout the universe. These particles are frequently subject to high-energy processes and subsequent condensation in the interstellar medium (ISM), where they are broken up into many ultrasmall silicate fragments. These abundant molecular-sized silicates likely play an important role in astrochemistry. By approximately mimicking silicate dust grain processing occurring in the diffuse ISM by ablation/cooling of a Mg/Si source material in the presence of O_2_, we observed the creation of stable clusters based on discrete pyroxene monomers (MgSiO_3_^+^), which traditionally have only been considered possible as constituents of bulk silicate materials. Our study suggests that such pyroxene monomer-based clusters could be highly abundant in the ISM from the processing of larger silicate dust grains. A detailed analysis, by infrared multiple-photon dissociation (IR-MPD) spectroscopy and density functional theory (DFT) calculations, reveals the structures and properties of these monomeric silicate species. We find that the clusters interact strongly with oxygen, with some stable cluster isomers having a silicate monomeric core bound to an ozone-like moiety. The general high tendency of these monomeric silicate species to strongly adsorb O_2_ molecules also suggests that they could be relevant to the observed and unexplained depletion of oxygen in the ISM. We further find clusters where a Mg atom is bound to the MgSiO_3_ monomer core. These species can be considered as the simplest initial step in monomer-initiated nucleation, indicating that small ionized pyroxenic clusters could also assist in the reformation of larger silicate dust grains in the ISM.

## 1 Introduction

Silicates constitute the main solid components of the universe. Terrestrially, silicates make up over 90% of the Earth’s crust and upper mantle and are also commonly found in rocky bodies throughout the Solar system and beyond. The formation of most silicates is thought to occur during high-temperature nucleation processes around dying stars,^1,2^ after which, they are ejected into the cold interstellar medium (ISM) as small dust grains. Generally, cosmic dust is thought to be hugely important for providing surface sites for astrochemical reactions, especially, but not only, when grains arrive in relatively denser and protected regions of the ISM.^3,4^ In the diffuse ISM, the nascent grains are subject to sporadic energetic processing by supernovae shockwaves, high-energy cosmic rays, and strong UV radiation, which lead to sputtering, shattering, and ionization before cooling again.^5,6^ Dust grain processing in the ISM is likely to create a huge population of nanosized silicate grains,^7^ which could be astrochemically relevant for formation/dissociation of H_2_^8,9^ and water molecules,^10,11^ and are a likely candidate for the source of the ubiquitous anomalous microwave emission.^12−14^ Silicate dust is mainly thought to be Mg-rich and of olivine (Mg_2_SiO_4_) or pyroxene (MgSiO_3_) composition^15^ and originally more olivinic when first formed.^16^ However, sputtering in the diffuse ISM leads to depletion of Mg cations,^17,18^ tending to relatively increase the fraction of pyroxenic dust.^19^ Due to the dominance of photoelectric electron ejection over recombination in very small grains in the diffuse ISM, nanosilicates are also likely to be positively ionized,^20^ which could enhance their role as seed species for nucleation.^21^

Here, we use laser ablation of a solid Si/Mg source material to produce thermally excited Mg and Si atomic/cationic species in the presence of O_2_, which then condense into small, ionized silicate clusters at lower temperature by collisional cooling with helium. In this way, we approximately mimic silicate dust grain processing conditions in the ISM. Our study identifies the detailed structures and infrared spectral characteristics of the produced cationic silicate clusters by comparing experimental infrared multiple-photon dissociation (IR-MPD) spectra with accurate quantum chemical calculations. We mainly focus on two distinct clusters, which we structurally determine to be based on the same cationic pyroxene monomer, MgSiO_3_^+^.

Studies of silicate cosmic dust nucleation have traditionally assumed that silicate monomers are only conceptually valid within bulk silicates and that they do not exist as stable discrete species.^22^ This assumption is based on considerations of molecular chemical stability in high-temperature (SiO/Mg/H_2_O)-based circumstellar silicate nucleation.^1,2^ Such a scenario is quite different from those of the ISM, where nanosilicates formed from dust processing events would be quickly cooled. Our work shows that cationic pyroxene monomers are structurally robust individual clusters that are readily produced in an ablation/cooling process. We further confirm that these species strongly interact with both oxygen and metal atoms/ions and thus could be chemically important species in astronomical environments.

## 2 Results and Discussion

### 2.1 IR-MPD Spectroscopy and Structure of MgSiO_9_^+^

Cationic magnesium silicate clusters were produced by pulsed laser ablation of a binary Mg_2_Si target in the presence of a 1% O_2_/He gas mixture that is subsequently expanded into vacuum. Mass spectrometric characterization of the formed clusters showed predominantly oxygen-rich clusters, Mg*_x_*Si*_y_*O*_z_*^+^ with *z* > *x* + *y*. Using isotopic labeling experiments with ^16^O_2_ and ^18^O_2_ and simulated isotopic distributions based on the natural abundance of Mg and Si, a series of magnesium oxides MgO_6,8,10_^+^ and Mg_2_O_5,7,9_^+^ as well as the magnesium silicates MgSiO_5,7,9,11_^+^ and Mg_2_SiO_7,9,11_^+^ were identified. Interestingly, no pure silicon oxide clusters Si*_y_*O*_z_*^+^ were observed, potentially due to the high magnesium content of the target (details on the experiment, mass spectra, and their analysis are given in Section S1).

**Figure 1:**
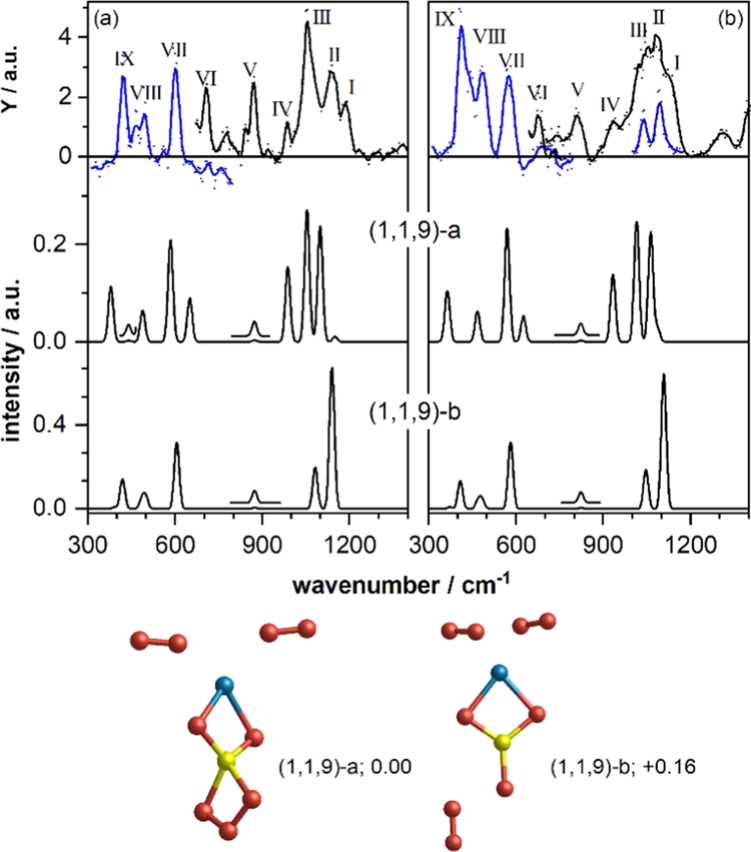
Top panels: IR-MPD spectra of (a) MgSi^16^O_9_^+^ and (b) MgSi^18^O_9_^+^. The dots represent the average of typically four to five spectra, and the solid lines represent a five-point average. The spectra in blue have been obtained at reduced IR macropulse intensity. Lower panels: calculated harmonic DFT spectra for the low-energy isomers (1,1,9)-a (middle panel) and (1,1,9)-b (bottom panel) with their respective structures shown below (relative energies in eV). The insets in the calculated spectra are magnified by a factor of 10. The labels (1,1,9) correspond to the nomenclature (*x*,*y*,*z*) for Mg*_x_*Si*_y_*O*_z_*^+^. Mg, Si, and O atoms are depicted as blue, yellow, and red spheres, respectively.

One of the most intense signals in the mass spectrum is observed at mass 196 amu, corresponding to MgSi^16^O_9_^+^ (cf. Section S2.1). Its infrared multiple-photon dissociation (IR-MPD) spectrum, recorded using the Free Electron Laser for Intra Cavity Experiments (FELICE),^23,24^ is shown in Figure 1a. The experimental spectrum shows nine bands in the 300–1400 cm^–1^ spectral region (labeled I–IX in Figure 1a, black spectrum). The IR-MPD spectrum for its MgSi^18^O_9_^+^ isotopologue (cf. Figure 1b) is very similar, although here bands I–III are less resolved. When irradiating this species at lower laser intensity, two well-separated bands remain (blue spectrum). Further bands observed are a doublet at 1585/1515 cm^–1^ (MgSi^16^O_9_^+^) and 1490/1430 cm^–1^ (MgSi^18^O_9_^+^), respectively, and one between 1900 and 2000 cm^–1^ (cf. Figure S7). The doublet is characteristic for the O–O stretch motion of weakly bound and rather unperturbed O_2_ units (compared to 1580 cm^–1^ for free ^16^O_2_),^25^ whereas the higher-frequency band is most likely a combination band. For clarity of comparison between the experimental and calculated spectra for the silicate cluster bands, we focus on the 300–1400 cm^–1^ spectral region in Figure 1.

Previous calculations of neutral pyroxene (MgSiO_3_)*_N_* clusters predicted the monomer (*N* = 1) to exhibit a rhombus-like MgSiO_2_ structure with the third oxygen atom terminally bound to the Si atom.^26^ The observation of the O–O stretch doublets in our spectra suggests that at least two oxygen atoms are bound as O_2_ molecules, but more could be possible. Thus, it seems reasonable to consider for MgSiO_9_^+^ structures containing a pyroxene (MgSiO_3_)-like core with three additional weakly bound oxygen molecules. Through using global optimization structural searches and density functional theory (DFT)-based calculations, such cluster cores were indeed found to be most energetically stable for MgSiO_3_^+^ (see Section S1.3 for computational methodology details), in which the unpaired spin is located on the terminal oxygen (cf. Figure S12). The relatively short Si–O bond length (1.57 Å) associated with this terminal silanone group indicates that it is a strong bond.^27,28^

However, in the lowest energy isomer found for MgSiO_9_^+^, the monomeric MgSiO_3_ core does not have a terminal oxygen atom, but possesses an O_3_ moiety forming a ring structure with the Si atom (isomer (1,1,9)-a in Figure 1). The remaining O atoms are in the form of two O_2_ molecules interacting with the chain-like MgSiO_5_ structure. Comparing DFT calculations of the bound O_3_ moiety and the free singlet ozone molecule (cf. Figure S13), we see that the former is somewhat more bent (+14°) and has a slightly longer O–O bond length (+0.1 Å). Like in ozone, the bound O_3_ species is polar with the central O atom having a similar positive charge. The terminal O atoms in the O_3_ species are less negatively charged than in free ozone because they bind to the Si atom of the pyroxene cluster core (see Figure S13). The two oxygen molecules are neutral and have triplet multiplicity. They are coordinated to the Mg atom with a 2.16 Å Mg–O distance. Curiously, the O_2_ inserted into the O_3_ unit and the O_2_ molecules interacting with the Mg atom all lead to a very similar energetic stabilization of the final system (∼0.6 eV) with respect to the separate relaxed MgSiO_3_^+^ cluster core. This energy is typical for a moderate nonbonding interaction, which is in line with the modest interaction between the O_2_ molecules and the Mg center. However, the intimate incorporation of O_2_ into the O_3_ unit clearly arises via the formation of new chemical bonds. The strength of the Si–O_3_ bonding is confirmed by considering the cluster core as an ozone molecule interacting with a MgSiO_2_^+^ cluster, where the respective binding energy is 4.3 eV. Overall, however, the energetic bonding stabilization in the MgSiO_5_^+^ core, with respect to the separated MgSiO_3_^+^ cluster and O_2_ molecule, is offset by the repulsive interaction between the positively polarized central oxygen of the O_3_ unit and the Si cationic center. This repulsion acts to weaken the two Si–O bonds with the O_3_ unit, which are ∼0.2 Å elongated relative to typical Si–O bonds (see the Supporting Information). We also note that the doublet spin multiplicity of the MgSiO_3_^+^ monomer is maintained in the MgSiO_5_^+^ core, albeit with some spin delocalization over the O_3_ unit.

The calculated vibrational spectrum of isomer (1,1,9)-a, displayed in the second panel of Figure 1, is in good agreement with the IR-MPD spectrum of MgSi^16^O_9_^+^. Although in the first instance, one is tempted to disqualify the match for lack of an intense counterpart for band I, a closer inspection shows that each of the bands labeled has a predicted counterpart. Bands I and II are well reproduced by calculated bands associated with a symmetric (1149 cm^–1^) and an asymmetric (987 cm^–1^) stretch vibration of the O_3_. These frequencies are relatively close to the observed values (1135 and 1089 cm^–1^)^29^ for free ozone, confirming the “ozonic” character of the O_3_ unit. Bands III and IV then correspond to the symmetric (1053 cm^–1^) and asymmetric (1098 cm^–1^) stretch motion of the OSiO unit in the MgSiO_2_ ring. A low-intensity band at 872 cm^–1^ corresponding to the O_3_ bending motion matches the observed frequency of band V, whereas the modes calculated at 651 cm^–1^ (OSiO bending mode of the MgSiO_2_ ring) and 585 cm^–1^ (OMgO symmetric stretch) are in agreement with bands VI and VII. Finally, bands VIII and IX are well reproduced by the calculated modes at 489 cm^–1^ (OMgO asymmetric stretch) and 380 cm^–1^ (MgSiO_2_ out of plane bending + MgSiO_3_–SiO_3_ stretch), respectively. Band VIII appears to have a low-frequency shoulder, which could reflect the calculated low-intensity mode at 441 cm^–1^ (asymmetric stretch of the OSiO unit of the SiO_3_ ring). This leaves only the small feature between bands V and VI, which cannot be explained by isomer (1,1,9)-a; we speculate that this may be a combination band. It should be mentioned that although all bands labeled I–IX are predicted in the calculated spectrum of isomer 1,1,9-a, their relative intensities are not always reflected well. In particular, bands I and V seem more intense than predicted, whereas the predicted intensity of band IV is higher than observed. This discrepancy is likely due to the multiple-photon absorption process in the experiment, for which intensities may deviate from those in the calculated linear absorption spectra.

Other isomers (1,1,9)-b to -d, each with three O_2_ units weakly bound to the MgSiO_3_^+^ cluster core, are found to be 0.16 eV, 0.20 eV, and 0.50 eV higher in energy, respectively (cf. Figures 1 and S6). These isomeric structures merely differ by the position of the three O_2_ units, which leads to very similar vibrational spectra; therefore, only the spectrum of isomer (1,1,9)-b is shown in Figure 1 (for others see Figure S6). The calculated spectra of all these isomers are in principle not in disagreement with the IR-MPD spectrum; however, none of them can explain all experimentally observed bands. In particular, none of them properly predicts the simultaneous observation of bands I and IV, which for isomer (1,1,9)-a are diagnostic for the O_3_ unit—a group not found in the other isomers. The O_3_ bending mode at 872 cm^–1^ is replaced by a mode containing components from the terminal Si–O stretch motion. Thus, for all isomers, a mode around 870 cm^–1^ is predicted, although of a different origin. The discrepancy between the observed strength for band I and its predicted intensity for isomer (1,1,9)-a may signal a minority population of isomer (1,1,9)-b.

To confirm the assignment of the experimental spectrum to isomer (1,1,9)-a, the IR-MPD spectrum of the isotopically labeled species MgSi^18^O_9_^+^ can be considered (cf. Figure 1b). Again, all features of the experimental spectrum are well reproduced by the calculated spectrum of isomer (1,1,9)-a, whereas the other isomers cannot account for all experimentally observed bands. It should be mentioned that the low-intensity mode calculated at 1149 cm^–1^ red-shifts upon isotopic labeling and overlaps with the neighboring band. Thus, instead of two separated bands, the first band is only slightly broadened on the blue side, which could explain the less resolved experimental features labeled I–III. Again, a (minor) contribution of isomer (1,1,9)-b cannot be ruled out.

Thus, it can be concluded the IR-MPD spectra of MgSi^16^O_9_^+^ and MgSi^18^O_9_^+^ are best described by isomer (1,1,9)-a, although small contributions of isomers (1,1,9)-b, (1,1,9)-c, and (1,1,9)-d cannot be completely excluded. Crucial is that the existence of a unique O_3_ unit is mandatory to satisfactorily explain all features of the experimental spectrum. The spectra of MgSiO_7_^+^ are very similar to those of MgSiO_9_^+^, indicating a similar structure of the cluster core with only one of the weakly bound O_2_ units removed (cf. Figure S8). The assignment of all calculated modes is summarized in Tables S1 and S2.

### 2.2 IR-MPD Spectroscopy and Structure of Mg_2_SiO_9_^+^

**Figure 2:**
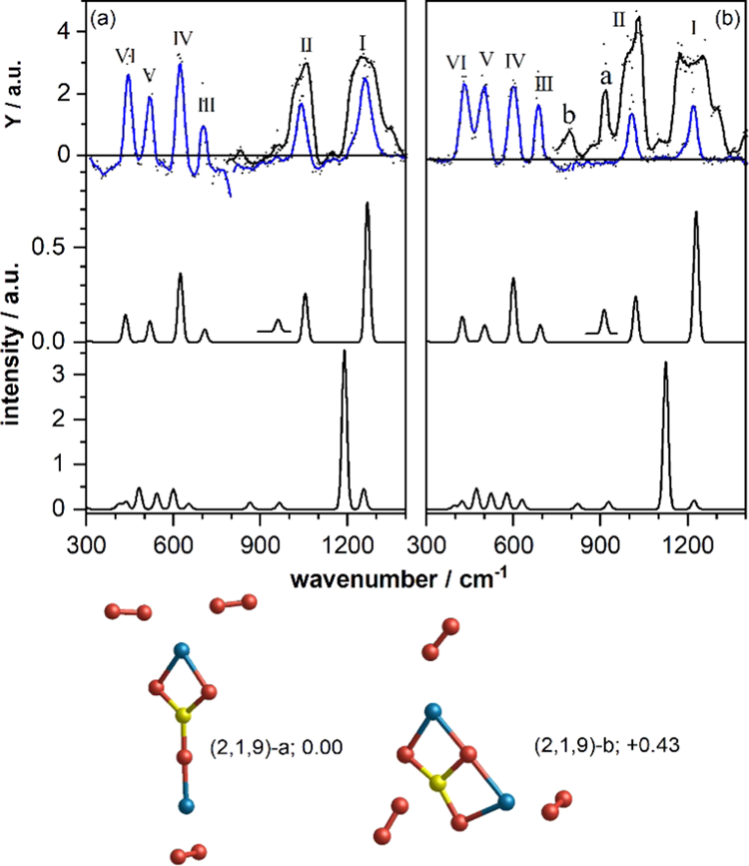
Top panels: IR-MPD spectra of (a) Mg_2_Si^16^O_9_^+^ and (b) Mg_2_Si^18^O_9_^+^. The dots represent the average of typically four to five spectra, and the solid lines represent a five-point average. The spectra in blue have been obtained at reduced IR macropulse intensity. Lower panels: calculated harmonic DFT spectra for the low-energy isomers (2,1,9)-a (middle panel) and (2,1,9)-b (lower panel) with the respective structures shown below (relative energies in eV). The insets in the calculated spectra of isomer (2,1,9)-a are magnified by a factor of 1000 (left) and 100 (right), respectively. The labels (2,1,9) correspond to the nomenclature (*x*,*y*,*z*) for Mg*_x_*Si*_y_*O*_z_*^+^. Mg, Si, and O atoms are depicted as blue, yellow, and red spheres, respectively.

A second intense peak in the mass spectrum (*m* = 220 amu) corresponds to Mg_2_Si^16^O_9_^+^ (cf. Figures S1 and S4). The IR-MPD spectrum for this mass (Figure 2a) and its O-18 isotopologue (Figure 2b) shows six well-resolved bands. Bands I and II are again recorded at lower laser intensity (blue spectrum), demonstrating that only band I is significantly broadened under normal irradiation conditions. For Mg_2_Si^18^O_9_^+^, irradiation under full laser intensity results in the appearance of two more bands labeled (a) and (b) in Figure 2b. One could speculate that these bands are also visible in Figure 2a, but the signal-to-noise ratio does not permit a conclusion. Bands I and II for both isotopologues are likely saturated under high-intensity irradiation, resulting in a broadening and, in particular band I for Mg_2_Si^18^O_9_^+^, a flattened-off peak signaling a full depletion of the ion population. As for MgSiO_9_^+^, two further bands (not shown here) are observed which can readily be assigned to the O–O stretch of an intact and quite unperturbed O_2_ unit and to a combination band (cf. Figure S10). To gain more insight into these features, calculated vibrational spectra must be considered.

The odd number of oxygen atoms in the cluster suggests that this cluster does most likely not have an olivine (Mg_2_SiO_4_)-like cluster core but one which is one oxygen atom deficient. Structures for neutral olivine nanoclusters (Mg_2_SiO_4_)*_N_* (i.e., with an even number of oxygen atoms) were reported previously,^26^ finding for *N* = 1 a Mg_2_SiO_3_ ring-like structure with the fourth oxygen atom terminally bound to the Si atom. This finding suggests that a similar ring-like structure could also exist for Mg_2_SiO_9_^+^.

However, the lowest energy structure found for Mg_2_SiO_9_^+^ consists of the same pyroxenic MgSiO_3_ core described above with an additional Mg atom terminally bound to the nonbridging O atom (isomer (2,1,9)-a in Figure 2). The remaining oxygen atoms form three O_2_ molecules, with two of them η^1^-bound to the Mg atom of the MgSiO_2_ ring and one η^2^-bound to the terminal Mg atom. The former are relatively weakly bound to the Mg_2_SiO_3_^+^ cluster core (0.47 eV) and can be regarded as distinct oxygen molecules. However, the latter O_2_ is strongly bonded to the terminal Mg atom (1.47 eV) with correspondingly short O–Mg distances (1.91 Å; cf. Figure S14). It also has a significantly elongated O–O bond length (1.33 Å) and a small excess negative charge, indicating that it has superoxide character. This character is also reflected in a strongly red-shifted vibrational frequency.

The calculated vibrational spectrum of isomer (2,1,9)-a (cf. Figure 2, second panel) shows six bands, which reproduce all bands labeled I–VI in the IR-MPD spectra of Mg_2_SiO_9_^+^. Band (a) for Mg_2_Si^18^O_9_^+^ might be explained by a very low intensity mode that becomes visible under 100-fold magnification. In contrast, for Mg_2_Si^16^O_9_^+^, this mode is predicted to be a factor of 10 weaker, which might explain the absence of this band in Figure 2a. Thus, merely band (b) in Figure 2b remains unexplained by isomer (2,1,9)-a. As for the unassigned band of MgSi^16^O_9_^+^ around 750 cm^–1^ (Figure 1a), we speculate that this is a combination band that becomes visible when irradiated at higher laser fluence.

A second structural motif found in the calculations is a T-shaped SiO_3_ with the two Mg atoms forming a rectangle (structures (2,1,9)-b in Figure 2 and (2,1,9)-c in Figure S9), which results in significantly different spectra. Promisingly, these isomers show two low-intensity bands between 900 and 1100 cm^–1^, which may account for bands (a) and (b). However, for isomer (2,1,9)-b, the strong O–O stretch of the η^2^-bound oxygen is red-shifted to 1189 cm^–1^, and no clear sign for this isomer is found experimentally. The strongest mode of isomer (2,1,9)-c, again the O–O stretch, but now of the η^1^-bound O_2_ molecule, is predicted at 1379 cm^–1^ for Mg_2_Si^16^O_9_^+^ and 1304 cm^–1^ for Mg_2_Si^18^O_9_^+^, also making it difficult to assign bands (a) and (b) to this species.

In summary, the vibrational spectrum of isomer (2,1,,9)-a is in favorable agreement with the IR-MPD spectrum of Mg_2_SiO_9_^+^ and accounts for all experimentally observed bands except for the low-intensity band (b). The assignment of all calculated modes is summarized in Tables S3 and S4. The IR-MPD spectra for Mg_2_SiO_7_^+^ (cf. Figure S14) are very similar to those of Mg_2_SiO_9_^+^, suggesting that this is a very similar cluster structure with one spectator O_2_ molecule less.

### 2.3 Implications for Interstellar Astrochemistry

As discussed above, ISM dust processing is consistent with the production of a high population of ultrasmall cationic pyroxenic species. Our experimental and theoretical infrared spectra could thus guide the possible observational identification of MgSiO_3_^+^-based clusters in the diffuse ISM by the James Webb space telescope (JWST).^30^ Previously, it has also suggested that pyroxene monomers could be detectable by high resolution microwave observations.^12,33^ If present, we further speculate that pyroxene monomer-based species could be relevant for astrochemical processes in the diffuse ISM.

From observations, it is found that the ISM is significantly depleted in oxygen relative to the expected abundance in typical dust grains (e.g., silicates, ices).^31^ For the denser regions of the ISM, where ice mantles start to grow on silicate grains, it has been suggested that highly hydroxylated nanosilicates could be one potential oxygen reservoir.^10^ For the diffuse ISM where only bare grains persist, our finding that cationic pyroxene monomers readily adsorb oxygen could point to a new reservoir for oxygen. The interaction strength of the oxygen molecules on the surface of amorphous silicates has been measured to be ∼0.08 eV and is likely to be too weak to provide a robust reservoir for oxygen in the harsh conditions of the diffuse ISM.^32^ However, with our cationic silicate monomer-based clusters, O_2_ molecules interact six to seven times more strongly (0.48–0.6 eV). For the (2,1,9)-a isomer of the Mg_2_SiO_9_^+^ cluster, we show that oxygen can bind even more strongly as superoxide-like O_2_ units. In addition to interactions with discrete O_2_ units, our lowest energy MgSiO_9_^+^ isomer (1,1,9)-a has an oxygen-rich core with a bound O_3_ unit and an overall O-to-metal ratio of 4.5 (2.5 in the MgSiO_5_ core). Compared with a maximum silicate dust O-to-metal ratio of 1.5 in stoichiometric pyroxene (MgSiO_3_), the potentially high abundance of cationic pyroxene monomers could play a significant role in explaining the missing oxygen in the diffuse ISM.

In the Mg_2_SiO_9_^+^ cluster, a Mg atom is bound to the MgSiO_3_ monomer core. This species can be considered as the simplest initial step in silicate grain growth starting from a monomeric seed cluster. Indeed, nucleation of metal atoms/ions onto grain seeds is thought to be one of the most important processes in dust grain (re)formation in the cool ISM.^21^ Our results provide tentative evidence that small ionized pyroxenic remnants of the dust destruction process could assist in the initial stages of silicate dust rebirth in the ISM.

## 3 Conclusions

We produced small silicate clusters from the ablation of a Mg_2_Si target in the presence of oxygen and subsequent cooling. We used IR-MPD spectroscopy and DFT calculations to obtain the IR spectra and structures of the species formed. We focus on two abundant clusters (MgSiO_9_^+^ and Mg_2_SiO_9_^+^), which are structurally found to be both based on the same MgSiO_3_^+^ pyroxene monomer core. In both cases, the cluster core is found to be interacting with six oxygen atoms. Four of the oxygen atoms in each case are associated with two oxygen molecules with a moderate nonbonding interaction with the respective cluster core. For MgSiO_9_^+^, the further two oxygen atoms form an ozone-like O_3_ unit, which is bonded to the cluster core. For the Mg_2_SiO_9_^+^, the remaining oxygen atoms are bound to the cluster core as a superoxide-like species. The cluster production method used is akin to high-energy processing of silicate dust grains in the ISM. The potential presence of cationic pyroxenic monomers in the ISM, together with their strong interaction species with oxygen and ozone, could have significant implications for, for example, the missing oxygen in the diffuse ISM and the initial stages of the silicate dust rebirth in the ISM. Our IR spectra for these new monomeric pyroxene based species could also be used as a reference for future JWST observations of nanosilicate dust in the ISM.^30^

## Supporting Information Available

Description of mass assignment, additional IR-MPD spectra, and assignment of experimentally observed bands; computational methodology and calculated structures, atom-partitioned charge, and spin populations of low energy clusters (PDF)

## Notes

In this work, no unexpected or unusually high safety hazards were encountered.
