# Supplementary material for: Cluster Beam Study of (MgSiO3)+-Based Monomeric Silicate Species and Their Interaction with Oxygen: Implications for Interstellar Astrochemistry
Source: ACS Earth Space Chem. 2022 Oct 6;6(10):2465–70. doi: 10.1021/acsearthspacechem.2c00186 (PMC9589904; doi:10.1021/acsearthspacechem.2c00186)
Supplement: Supplementary file 1 — sp2c00186_si_001.pdf [file sp2c00186_si_001.pdf]

Supporting Information for

Cluster Beam Study of $(MgSiO_3)^+$-based Monomeric Silicate Species and their Interaction with Oxygen: Potential Astronomical Relevance

Joan Mariñoso Guiu[1], Bianca-Andreea Ghejan,[2] Thorsten M. Bernhardt[2], Joost M. Bakker[3], Sandra M. Lang,[2]* Stefan T. Bromley[1,4]*

[1]Departament de Ciència de Materials i Química Física & Institut de Química Teòrica i Computacional (IQTCUB), Universitat de Barcelona, c/ Martí i Franquès 1-11, 08028 Barcelona, Spain

[2]Institute of Surface Chemistry and Catalysis, Ulm University, Albert-Einstein-Allee 47, 890691 Ulm, Germany

[3]Radboud University, Institute for Molecules and Materials, FELIX Laboratory, 6525 ED Nijmegen, The Netherlands

[4]Institució Catalana de Recerca i Estudis Avançats (ICREA), Passeig Lluis Companys 23, 08010 Barcelona, Spain

* Corresponding authors: sandra.lang@uni-ulm.de, s.bromley@ub.edu

S1 Methods

S1.1 Experimental method

Cationic magnesium silicate clusters were produced by pulsed laser ablation of a rotating MgSi rod target (Mg:Si = 2:1 molar ratio) using the second harmonic of a Nd:YAG laser. The ablation took place in a 3 mm diameter and 60 mm long growth channel in the presence of a short pulse of helium carrier gas seeded with 1% oxygen. The gas mixture was then expanded into vacuum forming a molecular beam, which was shaped by a 2 mm diameter skimmer and a 0.45 mm slit aperture, before entering the intracavity region where it interacted with the IR laser beam of the Free Electron Laser for Intra Cavity Experiments (FELICE, 310−2000 cm^{-1}; a 10 μs macropulse with picosecond duration micropulses at 1 GHz repetition rate; spectral width set to ~0.5% FWHM of the central frequency) crossing it at an angle of 35°. A few microseconds after interaction with FELICE, all ions were extracted by a set of pulsed high-voltage plates into a reflectron time-of-flight mass spectrometer and detected with a microchannel plate detector.[1,2]

To correct for long-term source fluctuations, the experiment was operated at twice the FELICE repetition rate, allowing for the recording of reference mass spectra in between successive FELICE pulses. Whenever the IR frequency was in resonance with an IR-active vibrational mode of a given complex,

multiple IR photons were absorbed sequentially, leading to a heating of the complex and finally to its fragmentation, and consequently to a depletion of the detected signal in the mass channel of the complex. The IR-MPD spectra shown in this contribution represent the depletion yield $Y(\tilde{\nu})$ at wavenumber $\tilde{\nu}$, obtained via the equation

$$Y(\tilde{\nu}) = -\frac{1}{P(\tilde{\nu})} \ln\left[\frac{I(\tilde{\nu})}{I_0}\right]$$

where $I(\tilde{\nu})$ and I_0 are the mass peak intensities with and without laser light, respectively, and $P(\tilde{\nu})$ is the macropulse energy. To reduce the IR fluence with which the complexes are irradiated, the whole instrument can be translated up to 300 mm from the focus position leading to a 30-fold reduction in fluence but increased overlap between the laser and molecular beam and thus an increased signal to noise ratio. All spectra presented in this work were recorded 300 mm from the focus. To reduce the IR intensity further, the overlap between laser and molecular beams can be purposely misaligned such that the molecular beam only observes the lower intensity part of the laser beam. Such a reduced IR intensity typically leads to an increased spectral resolution of strong vibrational transitions at the cost of no longer detecting weaker bands. To get the best possible spectral information, IR-MPD spectra were obtained under two different conditions with the molecular beam observing the high and the lower intensity part of the laser beam.

S1.2 Assignment of mass peaks

Laser ablation of a binary MgSi target in the presence of a 1% $^{16}O_2$/He pulse results in a typical distribution of magnesium silicate clusters as shown in Figure S1a. This mass spectrum is unusually rich due to the natural isotope distributions of magnesium and silicon, which leads to multiple mass peaks for each cluster size formed. Furthermore, 1% O_2 in the He carrier gas results in the preferred production of oxygen-rich clusters ($Mg_xSi_yO_z^+$ with z > x+y). Reduction of the oxygen percentage only reduces the overall cluster production but does not change the cluster size and composition distribution, showing the necessity of oxygen for the cluster formation process. Due to (1) the natural isotope distribution of Mg and Si which results in partially overlapping isotope distributions of different clusters and (2) the similar masses of Si_2 and MgO_2 units (m($^{28}Si_2$)=55.95 u and m($^{24}Mg^{16}O_2$)=55.97 u)[3] as well as O_3 and Mg_2 units (m($^{16}O_3$) = 47.98 u and m($^{24}Mg_2$)=47.97 u, a mass difference that cannot be resolved in the TOF mass spectrometer)[4] an unambiguous assignment of the individual mass peaks is not straightforward and must be done with particular care. Therefore, the experiments have been repeated with isotopically labeled oxygen ($^{18}O_2$) mixed into the He carrier gas as well with bare Si and Mg targets, respectively. The shift of individual mass signals upon isotopic labeling together with the calculated mass distribution arising (some examples are shown below) from the natural abundance of the Mg and Si isotope allows for a reliable assignment of the mass peaks. Furthermore, clusters of the same stoichiometry but produced with $^{16}O_2$ and $^{18}O_2$, respectively, are expected to exhibit similar patterns in the IR-MPD spectra with some spectral shifts due to the different masses of the oxygen isotopes. Comparison

with IR-MPD spectra obtained for clusters produced with bare Si and Mg targets, respectively, support or exclude the assignment to $Si_yO_z^+$ and $Mg_xO_z^+$ clusters. Based on all these considerations, we were able to unambiguously assign four series of clusters shown in Figure S1: the magnesium oxides $MgO_{6,8,10}^+$ and $Mg_2O_{5,7,9}^+$ as well as the magnesium silicates $MgSiO_{5,7,9,11}^+$ and $Mg_2SiO_{7,9,11}^+$. All other mass peaks cannot be assigned unambiguously and will not be further considered.

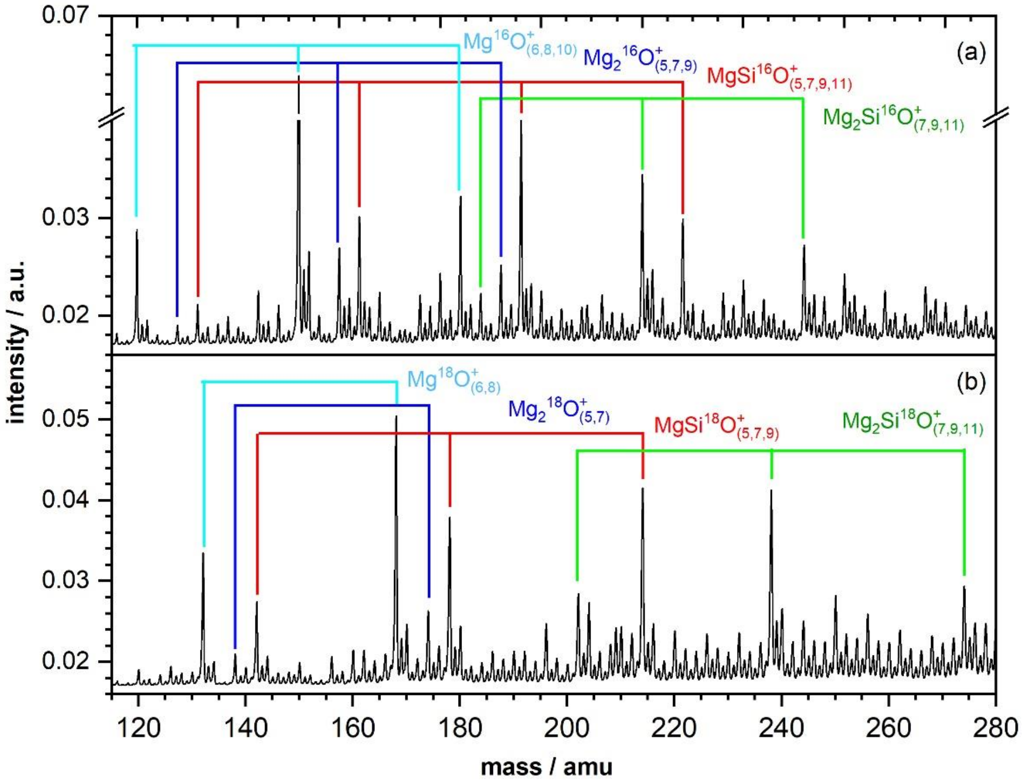

Figure S1. Ion mass distributions obtained by laser ablation of a Mg:Si = 2:1 target in the presence of a short pulse of helium carrier gas seeded with (a) 1% $^{16}O_2$ and (b) 1% $^{18}O_2$. The labeled mass signals were unambiguously assigned to the given stoichiometries based on isotope distribution patterns and the IR-MPD spectra.

S1.3 Computational methodology

To obtain different cluster isomers compatible with the experimental stoichiometries (see above) we initially assumed that clusters consist of a cationic bonded silicate core interacting with three oxygen molecules. We obtained candidate low energy isomers for each of the cores ($MgSiO_3$ and Mg_2SiO_3) using global optimization searches employing the Monte Carlo basin hopping algorithm[5] and a polarizable classical interatomic potential (IP) as detailed in previous studies.[6] For each silicate cationic core structure, we systematically sampled different positions around the core of the three interacting oxygen molecules. The structures of all candidates were optimized using Density Functional Theory (DFT) based calculations with the PBE0[7] hybrid exchange correlation functional, and an accurate tight-tier 2 numerical atom-centered orbital basis set.[8] Non-bonded dispersive interactions were described using the Tkatchenko-Scheffler method.[9] For all structures, harmonic IR spectra were calculated to confirm that

our structures correspond to true energy minima and for comparison with the experimental IR spectra. All DFT calculations were carried out with the Fritz Haber Institute Ab Initio Molecular Simulations package (FHI-AIMS).[10]

In addition to species with a core surrounded by separate O_2 molecules, we also tested configurations in which one or more oxygen molecules were more intimately interacting with the core and/or with other oxygen molecules. In all cases several spin states were evaluated. We note that in one of the low energy isomers reported we find that the monomeric pyroxene core is so intimately bound to an O_2 molecule that it forms a O_3 ozone-like moiety (unlike all other reported isomers in which the three O_2 molecules remain separate). We further note that DFT calculations using the PBE0 functional have been shown to be suitable for calculating the relative energetics of small oxide clusters interacting with oxygen in various ways with respect to benchmark renormalized second-order perturbation theory calculations.[11] The PBE0 functional was also shown to be suitable for calculating the energetics of O_3 interacting/reacting with a small molecule relative to multiconfigurational quantum chemical calculations.[12]

In our calculations we assume that the experimentally prepared clusters are cooled enough so that finite temperature anharmonic effects are minimal. Accurate high-level quantum chemical calculations of the neutral bare pyroxenic $MgSiO_3$ monomer have confirmed that anharmonicity hardly affects the pure harmonic IR spectrum.[13] Harmonic DFT calculations using the PBE0 functional have also been shown to accurately reproduce the infrared spectra of bulk crystalline olivine[14] and pyroxene[15], as well as the spectra of neutral olivinic and pyroxenic monomers.[16] We note that DFT calculations using the PBE0 functional overestimate the frequency of the stretching vibration for the free O_2 molecule by ~200 cm^{-1}. As the O-O stretch vibrations (1400 - 1600 cm^{-1}) associated with weakly bound O_2 molecules are spectrally well separated from the main signature silicate vibrations (300 - 1200 cm^{-1}), we mainly use the frequencies for the latter calculated using the PBE0 functional to identify experimentally produced isomers. As a separate theoretical check on the high frequency part of the experimental spectra we also performed vibrational calculations on all isomers using the PBE[17] functional which recovers the stretching vibrational frequency of the free O_2 molecule relatively well (although being poor for silicate vibrations).

S2 Assignment of mass peaks

S2.1 Assignment of mass m = 196 amu

One of the most intense signals in the mass spectrum obtained with a mixed MgSi target in the presence of helium seeded with $^{16}O_2$ is at mass m = 196 (cf. Figure S1). This mass can in principle be assigned to several different cluster stoichiometries, namely $Si_3{}^{16}O_7{}^+$, $MgSi^{16}O_9{}^+$, $Mg_3Si^{16}O_6{}^+$ or $Mg_5Si^{16}O_3{}^+$ (and some further oxygen-poor stoichiometries which we consider very unlikely to be produced). To determine the most likely stoichiometry, the experimentally detected isotope distribution (black curves in the

top panel of Figures S2) is compared with the isotope distribution of these clusters calculated on the basis of the natural abundance of Mg and Si (red curves in the top panel Figure S2). The best agreement between the experimental and the calculated isotope distribution is clearly given for $MgSi^{16}O_9^+$. To confirm this assignment, the experiments have been repeated with isotopically labeled oxygen, $^{18}O_2$ (Figure S2, bottom panel). The mass signal at m = 214 corresponding to $MgSi^{18}O_9^+$ is the only one that is comparable in intensity to the m = 196 amu signal in the $^{16}O_2$ experiment, whereas all other mass signals shown in the relevant mass spectral regions are considerably lower. The bottom panel of Figure S2 displays the theoretically obtained isotope distributions of $Si_3{}^{18}O_7^+$, $MgSi^{18}O_9^+$, $Mg_3Si^{18}O_6^+$ and $Mg_5Si^{18}O_3^+$ (green curves) together with the experimental mass spectra (black curves) in the corresponding mass regions. Again, the best agreement is obtained for $MgSi^{18}O_9^+$. Assignment of m = 196 amu in the $^{16}O_2$ experiments to the pure silicate cluster $Si_3O_7^+$ can be excluded upon comparison with the IR-MPD spectrum recorded on the mass of 196 amu produced with a pure Si target (in this case m = 196 amu can be unambiguously assigned to $Si_3{}^{16}O_7^+$) as shown in Figure S3. Finally, the assignment of m = 196 amu to $MgSi^{16}O_2^+$ is further substantiated by the similarity of the IR-MPD spectra (Figure 1 of the main text) recorded at m = 196 amu (with $^{16}O_2$) and m = 214 amu (with $^{18}O_2$), which show very similar patterns and spectral shifts consistent with the different masses of the oxygen isotopes.

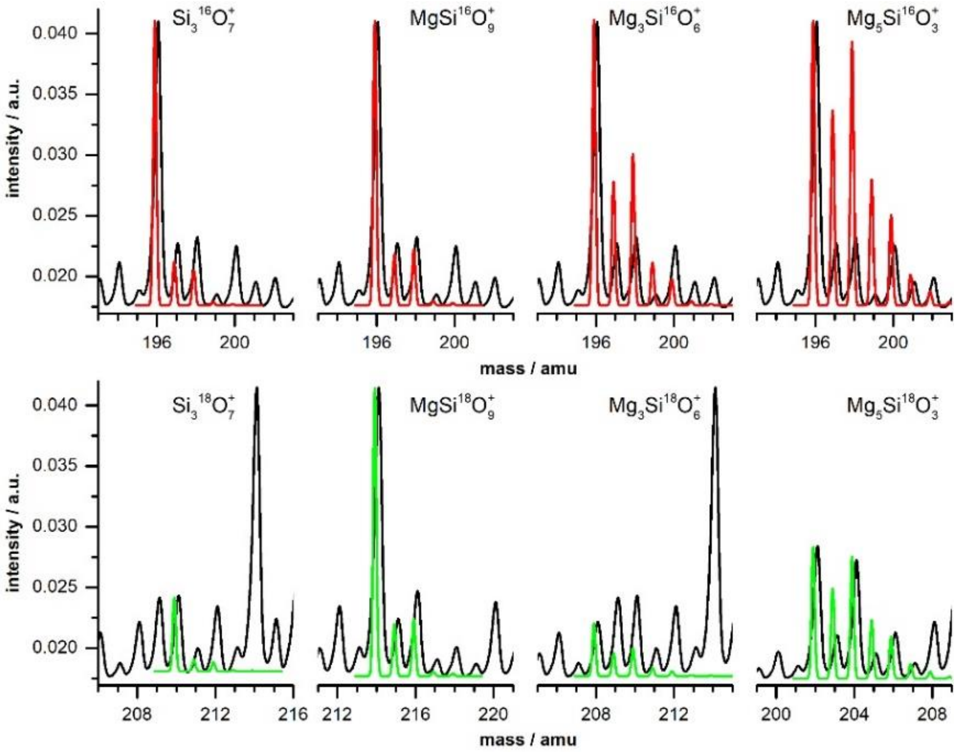

Figure S2: (top row) Experimentally obtained mass spectrum (black curves) around 196 amu obtained by laser ablation of a MgSi target in the presence of $^{16}O_2$. Also shown are theoretical isotope distributions (red curves) obtained on basis of the natural abundance of Mg and Si for several stoichiometries corresponding to a mass of 196 amu. (bottom row) Theoretical isotope distributions for the same clusters but with isotopically labeled $^{18}O_2$ (green curves) together with the experimental mass spectra in the corresponding mass regions.

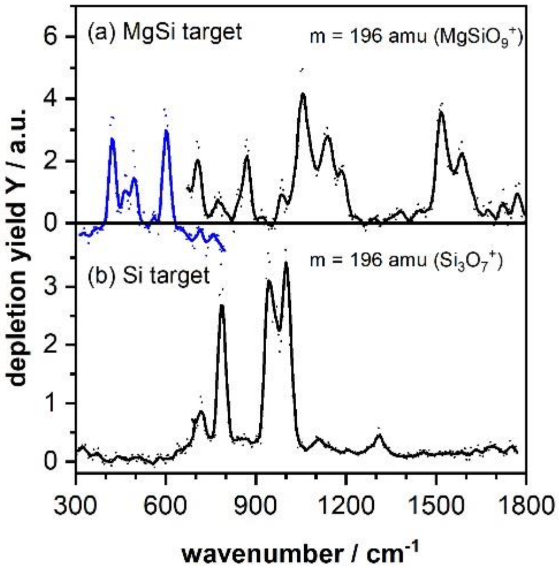

Figure S3: IR-MPD spectra obtained on the mass channel of 196 amu of the cluster distribution generated with a (a) mixed MgSi target and (b) a pure Si target, respectively. The mass m = 196 amu in the cluster distribution generated with a pure Si target (b) corresponds to the cluster stoichiometry $Si_3{}^{16}O_7{}^{+}$. In contrast, m = 196 amu in the cluster distribution obtained with a MgSi target (a) can in principle be assigned to different cluster stoichiometries as shown in Figure S2. Due to the significantly different IR-MPD spectra (a) and (b) an assignment to $Si_3{}^{16}O_7{}^{+}$ can be clearly excluded. The blue spectrum has been obtained at reduced IR macropulse intensity. The dots of the experimental spectra represent the sum of typically four to five spectra and the solid lines are obtained by a five-point average.

S2.2 Assignment of mass m = 220 amu

A second intense mass signal in the mass spectrum obtained with a mixed MgSi target in the presence of helium seeded with $^{16}O_2$ is at mass m = 220 amu (cf. Figure S1). This mass can in principle be assigned to $Si_5{}^{16}O_5{}^+$, $MgSi_3{}^{16}O_7{}^+$, $Mg_2Si^{16}O_9{}^+$, $Mg_4Si^{16}O_6{}^+$ or $Mg_6Si^{16}O_3{}^+$ (and again some further oxygen-poor stoichiometries which we deem very unlikely to be produced). To determine the most likely stoichiometry, the experimentally detected isotope distribution (black curves in the top panel of Figures S4) is compared with the isotope distribution of these clusters calculated on the basis of the natural abundance of Mg and Si (red curves in the top panel Figure S4). The best agreement between the experimental and the calculated isotope distribution is given for $Mg_2Si^{16}O_9{}^+$, but $MgSi_3O_7{}^+$ cannot be completely excluded based on these mass spectra alone. However, the mass spectrum obtained using $^{18}O_2$ shows only a very small signal at the mass of $MgSi_3{}^{18}O_7{}^+$, whereas the signal corresponding to the mass of $Mg_2Si^{18}O_9{}^+$ appears in comparable intensity as m = 220 amu. The calculated isotope distribution of $Mg_2Si^{18}O_9{}^+$ is also in favorable agreement with the experimentally observed isotope distribution.

Assignment of m = 220 amu to the pure silicate cluster $Si_5{}^{16}O_5{}^+$ can be excluded upon comparison of the IR-MPD spectra recorded for m = 220 amu produced with a pure Si target (in this case m = 220 amu can be unambiguously assigned to $Si_5{}^{16}O_5{}^+$) as shown in Figure S5. The assignment of m = 220 amu to $Mg_2Si^{16}O_9{}^+$ is confirmed beyond doubt by the similarity of its IR-MPD spectrum in comparison to the one recorded at m = 238 amu with $^{18}O_2$. Both spectra (cf. Figure 2 of the main text) show very similar patterns with some spectral shifts as expected to occur due to the different masses of the oxygen isotopes.

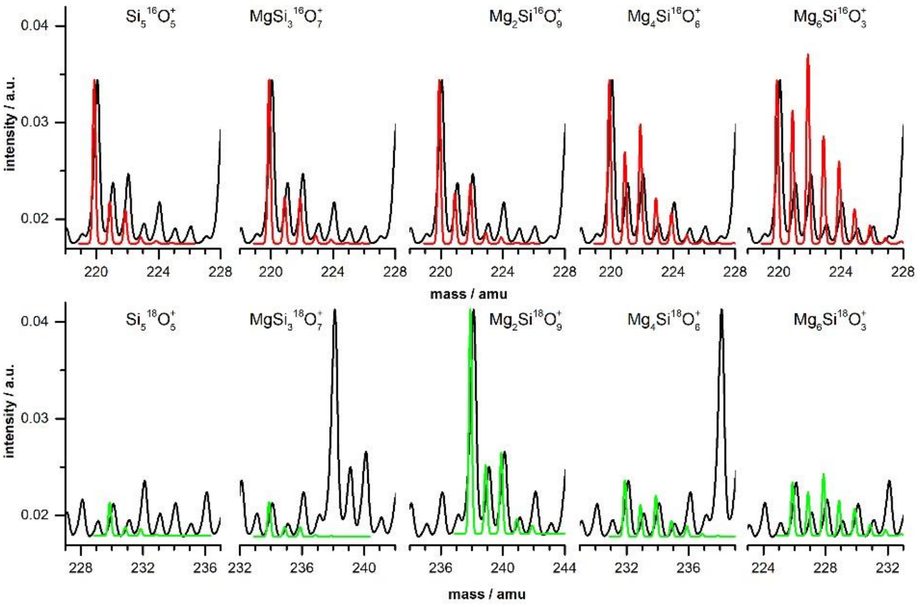

Figure S4: (top row) Experimentally obtained mass spectrum (black curves) around 220 amu obtained by laser ablation of a MgSi target in the presence of $^{16}O_2$. Also shown are theoretical isotope distributions (red curves) obtained on basis of the natural abundance of Mg and Si for several stoichiometries corresponding to a mass of 220 amu. (bottom row) Theoretical isotope distributions for the same clusters but with isotopically labeled $^{18}O_2$ (green curves) together with the experimental mass spectra in the corresponding mass regions.

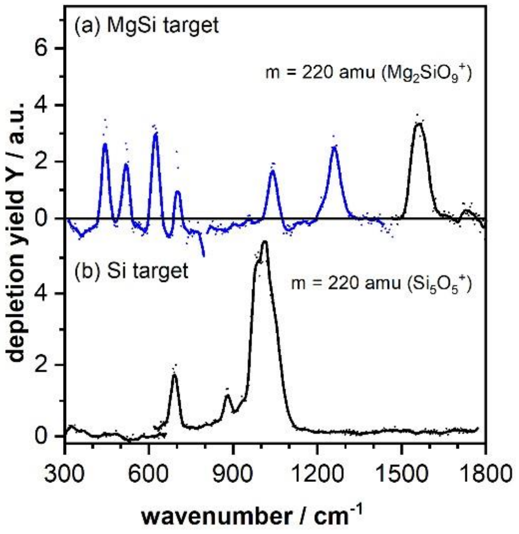

Figure S5: IR-MPD spectra obtained on the mass channel of 220 amu of the cluster distribution generated with a (a) mixed MgSi target and (b) a pure Si target, respectively. The mass m = 220 amu in the cluster distribution generated with a pure Si target (b) corresponds to the cluster stoichiometry $Si_5{}^{16}O_5{}^+$. In contrast, m = 220 amu in the cluster distribution obtained with a MgSi target (a) can in principle be assigned to different cluster stoichiometries as shown in Figure S4. Due to the significantly different IR-MPD spectra (a) and (b) an assignment to $Si_5{}^{16}O_5{}^+$ can be clearly excluded. The blue spectrum has been obtained at reduced IR macropulse intensity. The dots of the experimental spectra represent the sum of typically four to five spectra and the solid lines are obtained by a five-point average.

S3 Additional vibrational spectra and mode assignment

S3.1 IR-MPD spectra of $MgSiO_9{}^+$

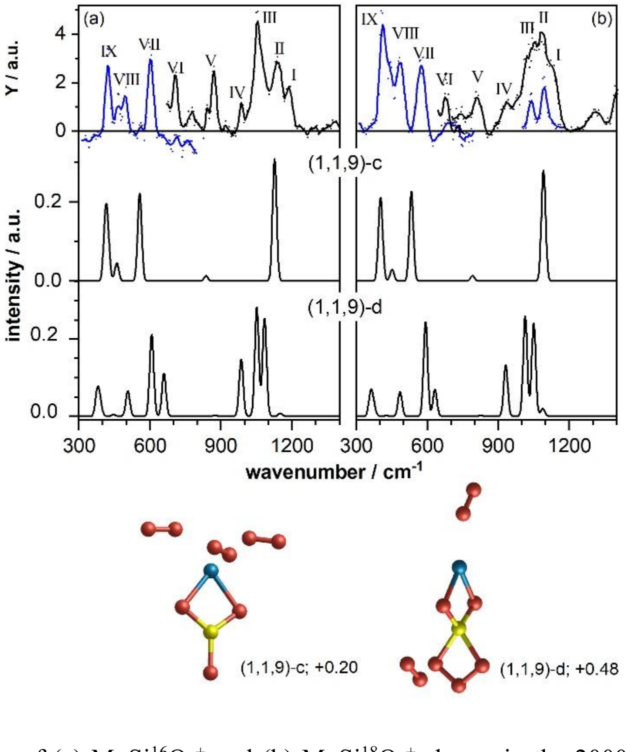

Figure S6: IR-MPD spectra of (a) $MgSi^{16}O_9{}^+$ and (b) $MgSi^{18}O_9{}^+$ shown in the 2000-1300 cm^{-1} spectral region together with the PBE0-calculated vibrational spectra of isomers (1,1,9)-c and (1,1,9)-d. The spectra of other iso-

meric structures are shown in the main text. The labels (x,y,z) correspond to $Mg_xSi_yO_z^+$. The dots of the experimental spectra represent the sum of typically four to five spectra and the solid lines are obtained by a five-point average. Mg, Si, and O atoms are depicted as cyan, yellow, and red spheres, respectively.

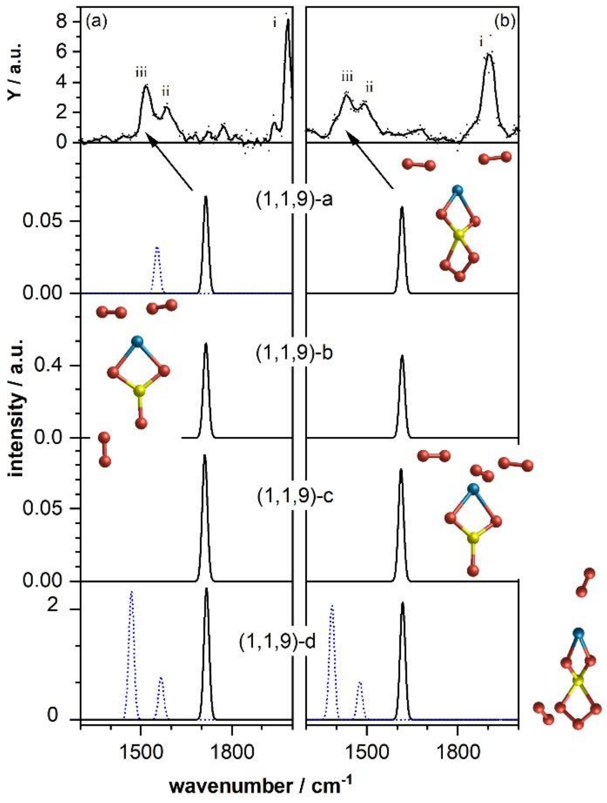

Figure S7: IR-MPD spectra of (a) $MgSi^{16}O_9^+$ and (b) $MgSi^{18}O_9^+$ shown in the 2000-1300 cm^{-1} spectral region together with the calculated vibrational spectra of several isomeric structures. The black and dotted blue spectra were calculated using the PBE0 and PBE functionals, respectively. The black spectra for isomer (1,1,9-d) are magnified by a factor of 50. The spectra in the 1400-300 cm^{-1} region are discussed in the main text. The labels (x,y,z) correspond to $Mg_xSi_yO_z^+$. The dots of the experimental spectra represent the sum of typically four to five spectra and the solid lines are obtained by a five-point average. Mg, Si, and O atoms are depicted as cyan, yellow, and red spheres, respectively.

The IR-MPD spectra of $MgSi^{16}O_9^+$ and $MgSi^{18}O_9^+$ in the 2000-1300 cm^{-1} spectral region (Figure S7) both show a double peak (labeled ii and iii) at 1585/1515 cm^{-1} ($MgSi^{16}O_9^+$) and 1490/1430 cm^{-1} ($MgSi^{18}O_9^+$), respectively. The frequencies of these bands are indicative for the O-O stretch of an almost unperturbed O_2 unit (compare to 1580 cm^{-1} for free $^{16}O_2$),[18] and their average frequency reduction by a factor 0.942 upon isotopic substitution is close to the textbook ratio of 0.943 for a diatomic O-O stretch. The double peak structures indicate an isomer with two differently bound O_2 units or two isomeric structures, each with differently bound oxygen molecules. In the structures calculated, either two (isomers (1,1,9)-a,d) or three (isomers (1,1,9)-b,c) O_2 molecules are bound at different positions. As noted above, our PBE0-based DFT calculations systematically overestimate the O_2 stretching frequencies and predict that they are almost unchanged for all O_2 molecules. Tests for the O_2-based frequencies using PBE-based DFT calculations show an expected downshift of the O-O stretching modes to the experimentally observed range. Moreover, the PBE-based calculations also indicate that symmetrically inequivalent positions for bound O_2 molecules give rise to distinct peaks in good agreement with the experimentally observed double peak (see example for isomer (1,1,9)-d). We note that isomer (1,1,9)-d

has the same silicate core as (1,1,9)-a and may indicate that different positions of relatively "free" O_2 molecules are sampled around this $MgSiO_5$ core species during the experiment.

Near the blue-edge of the $MgSi^{16}O_9^+$ spectrum, a final unexplained band (labeled i) is observed. This band red-shifts considerably to around 1900 cm^{-1} upon isotopic labelling. Since this spectral region is neither characteristic for Mg-O and Si-O nor for O-O vibrations, this band cannot arise from a fundamental vibrational motion. We speculate that this band may be assigned to a combination band between the O-O stretch (ii and iii) vibrations and the two bands labeled VIII and IX (cf. Figure 1 in the main text). Both the ii/IX and the iii/VIII combination can lead to the frequency of band i; this frequency coincidence of both combinations may explain the unexpectedly high intensity for a combination band with which this band is observed.

Table S1: Frequencies of the observed bands in the IR-MPD Spectrum of $MgSi^{16}O_9^+$, those of the assigned calculated bands for isomer (1,1,9)-a, and their mode descriptions.

**experimental band**	**experimental frequencies**	**calculated frequencies for isomer (1,1,9)-a**	**mode[(a)]**
I	1185	1149	O_3 unit symmetric stretch<br>Si-O stretch (terminal O)
II	1137	1098	OSiO (of $MgSiO_2$ ring) asymmetric stretch
III	1060	1053	OSiO (of $MgSiO_2$ ring) symmetric stretch
IV	987	987	O_3 unit asymmetric stretch
V	870	872	O_3 unit bending
VI	706	651	OSiO (of $MgSiO_2$ ring) bending
VII	602	585	OMgO symmetric stretch
VIII	493	489	OMgO asymmetric stretch
shoulder of VIII	468	441	OSiO (of SiO_3 ring) asymmetric stretch
IX	423	380[(b)]	$MgSiO_2$ out of plane bending / $MgSiO_2$-SiO_3 symmetric stretch

[(a)] Most of the normal modes are coupled modes containing contributions from two or more motions involving several atoms, which makes mode assignments usually difficult. The given mode assignment is based on the most prominent motions, but additional other atoms are typically also involved. [(b)] two modes with almost the same frequency.

Table S2: Frequencies and mode descriptions of the main calculated bands for isomers (1,1,9)-b, (1,1,9)-c, and (1,1,9)-d.

**isomer (1,1,9)-b**	**isomer (1,1,9)-c**	**isomer (1,1,9)-d**	**mode[(a)]**
--	--	--	
1139	1128	1133	Si-O stretch (terminal O) + OSiO (of $MgSiO_2$ ring) symmetric stretch
	1126		OSiO (of $MgSiO_2$ ring) asymmetric stretch
1081	--	1060	OSiO (of $MgSiO_2$ ring) asymmetric stretch
--	--	--	
873[(a)]	836	868	OSiO (of $MgSiO_2$ ring) symmetric stretch + Si-O (terminal O) stretch
--	--	--	

606	557	626	OMgO symmetric stretch
499		515	OMgO asymmetric stretch
486[b]	460	498[a]	Mg-Si stretch
420	412	409	$MgSiO_2$ out of plane bending
392		395	O-O_2 stretch
	422		OMgO asym stretch

[a] Most of the normal modes are coupled modes containing contributions from two or more motions involving several atoms, which makes mode assignments usually difficult. The given mode assignment is based on the most prominent motions but additional other atoms are typically also involved. [b] combined with OMgO asymmetric stretch

S3.2 IR-MPD spectra of $MgSiO_z^+$ (z = 7,9)

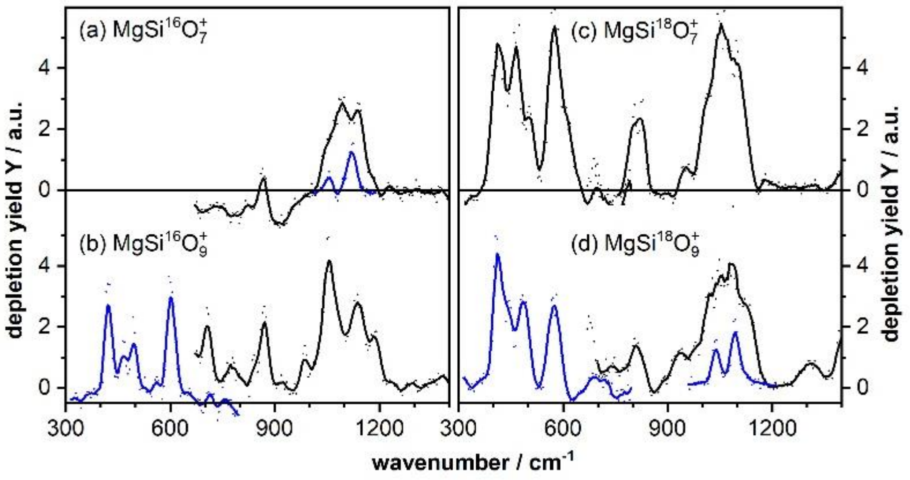

Figure S8: IR-MPD spectra of (a) $MgSi^{16}O_7^+$, (b) $MgSi^{16}O_9^+$ as well as the isotopically labelled counterparts (c) $MgSi^{18}O_7^+$ and (d) $MgSi^{18}O_9^+$. The spectra of $MgSi^{16}O_9^+$ and $MgSi^{18}O_9^+$ are discussed in detail in the main text. The IR-MPD spectra of $MgSi^{16}O_7^+$ and $MgSi^{18}O_7^+$ show similar modes as the spectra of $MgSi^{16}O_9^+$ and $MgSi^{18}O_9^+$ indicating a similar cluster core. The low wavenumber region of the IR-MPD spectrum of $MgSi^{16}O_7^+$ is dominated by signal gain and is thus not shown here. The blue parts of the spectra have been obtained at reduced IR macro-pulse intensity. The dots of the experimental spectra represent the sum of typically four to five spectra and the solid lines are obtained by a five-point average.

S3.3.1 IR-MPD spectra of $Mg_2SiO_9^+$: alternative isomers.

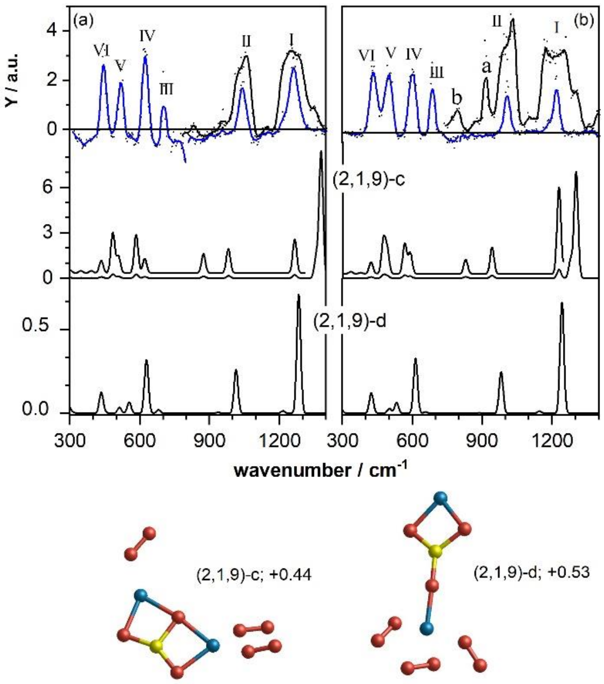

Figure S9: IR-MPD spectra of (a) $Mg_2Si^{16}O_9^+$ and (b) $Mg_2Si^{18}O_9^+$ shown in the 2000-1300 cm^{-1} spectral region together with the calculated vibrational spectra of isomers (2,1,9)-c and (2,1,9)-d. The spectra of other isomeric structures are shown in the main text. The labels (x,y,z) correspond to $Mg_xSi_yO_z^+$. The dots of the experimental spectra represent the sum of typically four to five spectra and the solid lines are obtained by a five-point average. Mg, Si, and O atoms are depicted as cyan, yellow, and red spheres, respectively.

S3.3.2 IR-MPD spectra of $Mg_2SiO_9^+$: alternative isomers in the high-frequency range.

The IR-MPD spectra of $Mg_2Si^{16}O_9^+$ and (b) $Mg_2Si^{18}O_9^+$ in the 2000-1300 cm^{-1} spectral region are shown in Figure S10. Both spectra show one intense band (labeled ii) around 1560/1480 cm^{-1} (for $Mg_2Si^{16}O_9^+$/$Mg_2Si^{18}O_9^+$), and one lower intensity band (i) around 1970/1900 cm^{-1}. The high intensity bands are indicative for the O-O stretch of an almost unperturbed O_2 unit (compared to 1580 cm^{-1} for free $^{16}O_2$).[18] The geometry of the calculated isomers (2,1,9)-a and (2,1,9)-d are very similar with the O_2 units bound at different positions. However, due to the rather large distance of the O_2 to the cluster core (> 2.1 Å), the position hardly effects the frequency of the O-O stretch mode, which is calculated to be between 1715 and 1700 cm^{-1} for both isomers. In contrast, isomer (2,1,9)-b contains two non-identical O_2 units. The distance between the Si atom and the O_2 is with 2.4 Å rather large leading to a very low intensity mode at 1695 cm^{-1}. The distance between the Mg atom and the second O_2 is considerably shorter (2.04 Å), which results in a red-shift of the mode to 1561 cm^{-1} and a considerably increased intensity. Finally, in isomer (2,1,9)-c the two additional oxygen atoms are almost identical and are both bound to the same Mg atom. This leads to a very low intensity mode at 1552 cm^{-1}. The intense mode predicted at 1379 cm^{-1} corresponds to the O-O stretch of the third O_2 unit η^1-bound to the Mg atom. All

the discussed O-O bands (except for the 1379 cm^{-1} mode of isomer (2,1,9)-c) can account for the experimentally observed band (ii). The mode labeled (i) can, similarly to the above discussed case of $MgSiO_9^+$, not be explained by fundamental vibrational modes but must instead be assigned to a combination band.

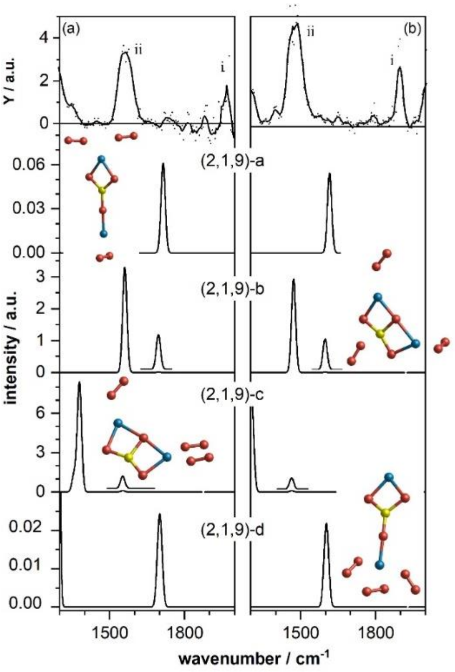

Figure S10: IR-MPD spectra of (a) $Mg_2Si^{16}O_9^+$ and (b) $Mg_2Si^{18}O_9^+$ shown in the 2000-1300 cm^{-1} spectral region together with the calculated vibrational spectra of several isomeric structures. The spectra in the 1400-300 cm^{-1} region are discussed in the main text. The labels (x,y,z) correspond to $Mg_xSi_yO_z^+$. The insets in the calculated spectra of isomer (2,1,9)-c are magnified by a factor of 10, those of isomer (2,1,9)-b by a factor of 100. The dots of the experimental spectra represent the sum of typically four to five spectra and the solid lines are obtained by a five-point average. Mg, Si, and O atoms are depicted as cyan, yellow, and red spheres, respectively.

Table S3: Assignment of bands (labeled I−VI) observed in the IR-MPD Spectrum of $Mg_2Si^{16}O_9^+$ shown in Figure 2a of the main text and S9. The bands are assigned based on calculated vibrational spectra of isomers (2,1,9)-a and (2,1,9)-b.

**experimental band**	**experimental frequencies**	**calculated frequencies for isomer (2,1,9)-a**	**calculated frequencies for isomer (2,1,9)-d**	**mode[(a)]**
I	1261	1268	1283	Si-O stretch
		1208	1215	O-O (of η^2-bound O_2) stretch
II	1041	1054	1014	OSiO (of $MgSiO_2$ ring) asymmetric stretch
III	704	780	--	OSiO (of $MgSiO_2$ ring) + Mg-O_2 symmetric stretch
IV	624	624	680,628	OMgO (of $MgSiO_2$ ring) symmetric stretch + Mg-O_2 symmetric stretch
V	518	521	555	OMgO (of $MgSiO_2$ ring) asymmetric stretch
		517	513	Mg-Si stretch
		488	--	Mg-O_2 asymmetric stretch
VI	445	435	434	$MgSiO_2$ out of plane bend
			451	Mg-O_2 asymmetric stretch
a		960	935	OSiO (of $MgSiO_2$ ring) symmetric stretch
b				

[(a)] Most of the normal modes are coupled modes containing contributions from two or more motions involving several atoms, which makes mode assignments usually difficult. The given mode assignment is based on the most prominent motions, but additional other atoms are typically also involved.

Table S4: Assignment of bands (labeled I−VI) observed in the IR-MPD Spectrum of $Mg_2Si^{16}O_9^+$ shown in Figure S9. The bands are assigned based on the calculated vibrational spectrum of isomer (2,1,9)-c.

**experimental band**	**isomer (2,1,9)-c**	**mode[(a)]**
I	1379	O-O (of η^1-bound O_2) stretch
	1358	O-O (of two Mg η^1-bound O_2) stretch
	1266	O(2)SiO(3) asymmetric stretch
II	--	
III	--	
IV	621	O(2)Mg(1)O(1) symmetric stretch
	585	O(3)Mg(2)O(1) symmetric stretch
V	509	O(2)SiO(3) bend + Mg(1)-O_2 symmetric stretch
	485	Mg-Si stretch
VI	435	Mg_2SiO_3 out of plane bend
a	981	O(2)SiO(3) symmetric stretch
b	874	Si-O(1) stretch

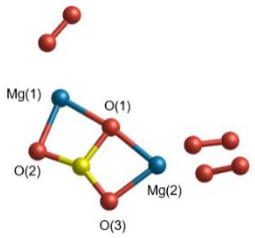

[(a)] Most of the normal modes are coupled modes containing contributions from two or more motions involving several atoms, which makes mode assignments usually difficult. The given mode assignment is based on the most prominent motions but additional other atoms are typically also involved. [(b)] two modes with almost the same frequency

S3.4 Vibrational Spectra of $Mg_2SiO_z^+$ (z = 7,9)

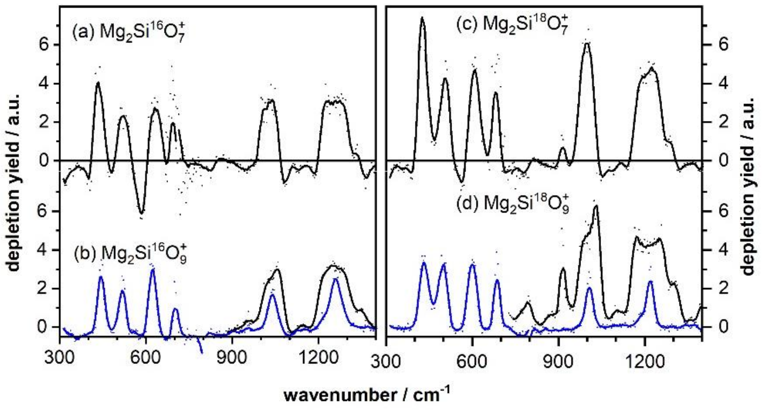

Figure S11: IR-MPD of (a) $Mg_2Si^{16}O_7^+$, (b) $Mg_2Si^{16}O_9^+$ as well as the isotopically counterparts (c) $Mg_2Si^{18}O_7^+$ and (d) $Mg_2Si^{18}O_9^+$. The spectra of $Mg_2Si^{16}O_9^+$ and $Mg_2Si^{18}O_9^+$ are discussed in detail in the main text. The IR-MPD spectra of $Mg_2Si^{16}O_7^+$ and $Mg_2Si^{18}O_7^+$ show similar modes as the spectra of $MgSi^{16}O_9^+$ and $MgSi^{18}O_9^+$ indicating a similar cluster core. The blue parts of the spectra have been obtained at reduced IR macropulse intensity. The dots of the experimental spectra represent the sum of typically four to five spectra and the solid lines are obtained by a five-point average.

S4 Structures and charge/spin populations of selected low energy clusters

S4.1 $MgSiO_3^+$ cluster core

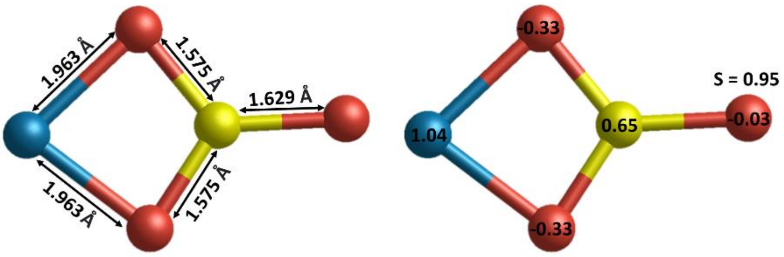

Figure S12: Structure and charge/spin populations of $MgSiO_3^+$. The numbers in left structure denote bond lengths and the numbers in the right structure denote charges. Also given is the spin (S) on the terminal oxygen atom. Mg, Si, and O atoms are depicted as blue, yellow, and red spheres, respectively.

S4.2 Isomer (1,1,9)-a of the $MgSiO_9^+$ cluster

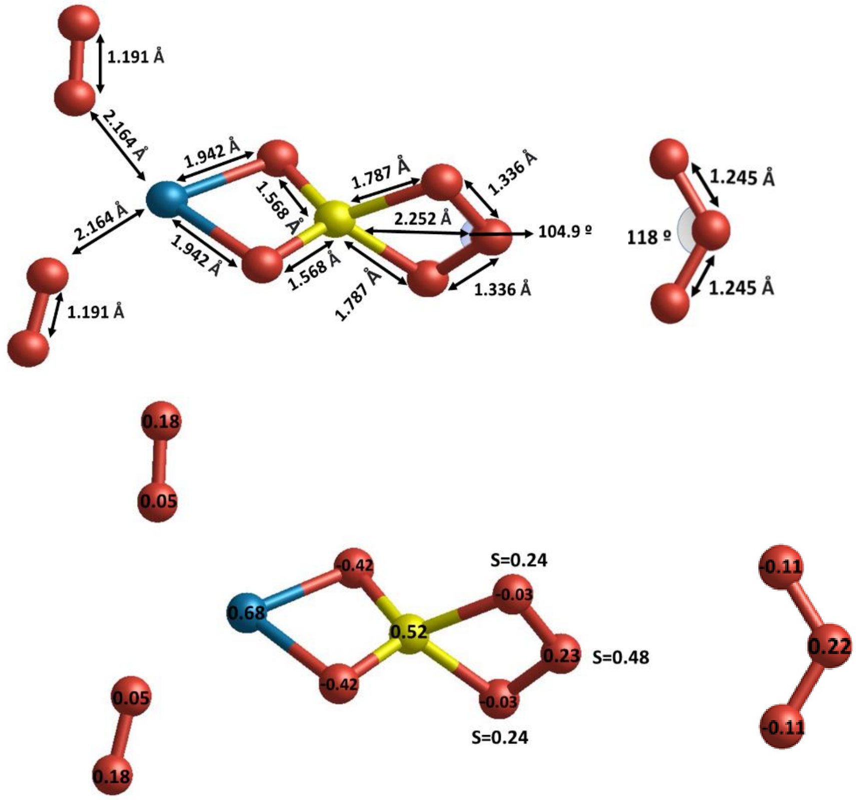

Figure S13: Structure and charge/spin populations of $MgSiO_9^+$ (left) in comparison with free ozone O_3 (right). The numbers in the top structure denote bond lengths and the numbers in bottom structures denote charges. We also provide the atomically partitioned spin populations (S) on the oxygen atoms of the bound O_3 unit. Mg, Si, and O atoms are depicted as blue, yellow, and red spheres, respectively.

S4.3 Isomer (2,1,9)-a of the $Mg_2SiO_9^+$ cluster

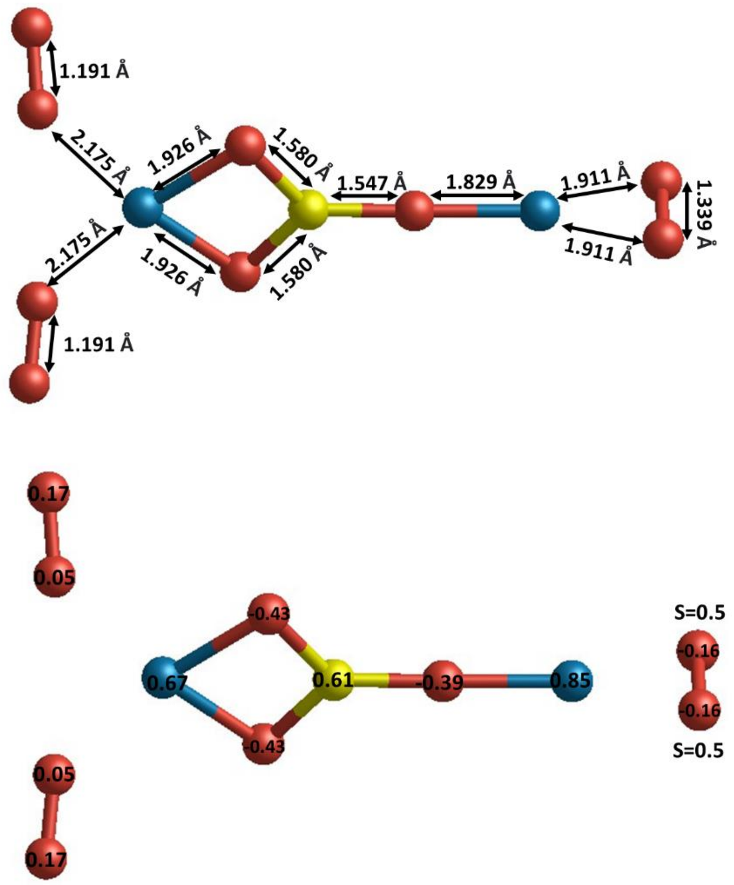

Figure S14: Structure and charge/spin populations of $Mg_2SiO_9^+$. The numbers in the top structure denote bond lengths and the numbers in the bottom structure denote charges. We also provide the atomically partitioned spin populations (S) on the oxygen atoms of the O_2 molecule bound to the terminal Mn atom. Mg, Si, and O atoms are depicted as blue, yellow, and red spheres, respectively.

S4.4 Cartesian coordinates of $MgSiO^+$ and $Mg_2SiO_9^+$ clusters.

Below we include the cartesian coordinates of a selection of low energy clusters from our DFT calculations. The data is given using the xyz file format where the first line gives the number of atoms in the system, the second line gives the relative total energy with respect to the respective lowest energy and the remaining lines provide the optimized cartesian coordinates of each atom.

(1,1,9)-a

11			
0 eV			
Mg	-1.93284741	2.99401786	1.28533342
O	-0.29591504	3.91841519	0.79644574
O	-0.65696986	1.57039482	1.63041566
Si	0.44393573	2.58677278	1.16824778
O	1.9669517	2.68631486	2.09692686
O	2.66163727	2.20976175	1.05991453
O	1.7532948	2.00514873	0.10148242
O	-3.6671923	2.24623932	0.23051686
O	-3.99460305	1.22050613	-0.27807524
O	-3.19619987	4.26048074	2.50364762
O	-3.08876195	5.31752783	3.04152435

(1,1,9)-b

11			
0.16 eV			
Mg	-2.83758391	2.68252166	0.37347008
O	-1.58187134	4.06834407	-0.13778206
O	-1.36099991	2.20424879	1.54167524
Si	-0.59990307	3.42879545	0.9097142
O	0.87064301	3.93745406	1.24710188
O	1.62736195	2.75330997	2.63410995
O	-4.81653672	3.51139366	0.65495712
O	-3.66978865	0.95318192	-0.62807551
O	-3.37839431	-0.19284522	-0.76721175
O	-5.28961783	4.60317171	0.70166484
O	2.72258078	3.08288395	2.84725601

(1,1,9)-c

11			
0.20 eV			
Mg	-1.80140211	2.50174372	0.51703798
O	-0.28284108	1.3462982	-0.00017021
O	-0.26761506	3.63108084	1.08309009
Si	0.63828665	2.47648543	0.55467435
O	2.25631427	2.45672234	0.57771774
O	-2.82448551	3.11238021	3.42528978
O	-2.97510658	2.56472373	2.37825958
O	-2.95288473	4.05493575	-0.53716558
O	-2.78558875	5.22289201	-0.70102084
O	-3.2903986	0.03734609	-0.72079237
O	-3.35502853	1.11484167	-0.21717049

(1,1,9)-d

11
0.39 eV

Mg	-1.48324688	2.30055321	1.14412419
O	-0.11098692	3.13614015	0.08042807
O	0.05608934	1.49203499	1.97324465
Si	0.91361021	2.32369511	0.95224169
O	2.38739255	3.14925546	1.52415040
O	3.15311907	2.36218111	0.76360203
O	2.29687110	1.54656637	0.14078684
O	-4.54723559	2.95174385	0.73306551
O	-3.59577379	2.43479197	1.22819919
O	2.98013873	0.32448356	3.14318612
O	2.39980219	-0.35168578	3.93437234

(2,1,9)-a

12
0 eV

O	-0.84486215	5.15311159	-0.10506699
O	-1.29469365	3.41902408	1.65157813
O	1.30505707	3.50011438	0.60701055
Si	-0.16021639	3.98474234	0.70859529
Mg	-2.45378611	4.74568577	0.87288489
Mg	3.03516706	2.91510293	0.50829379
O	-3.63980886	5.71966294	2.41360306
O	-3.67030728	5.69679339	3.60377969
O	-4.18104363	4.72242801	-0.44729602
O	-4.35193573	4.77610124	-1.62454014
O	4.91083853	2.80536645	0.86032574
O	4.5431211	1.86649689	-0.020898

(2,1,9)-b

12
0.43 eV

O	-0.46312124	5.73848497	0.94198145
O	-2.32663145	4.0580926	0.68539056
O	-0.34735961	4.79286619	-1.42392764
Si	-1.06251298	4.59283325	-0.06735
Mg	-1.957149	5.29871587	2.10894376
Mg	0.69117235	6.30919875	-0.69324248
O	-2.67247542	5.92401964	3.91317617
O	-2.44043902	6.79540577	4.71556983
O	1.40610635	8.09844665	-1.00236521
O	2.37537517	7.28966059	-0.63738633
O	0.32058806	2.76240887	0.58843087
O	0.56504678	1.82902687	-0.11114095

(2,1,9)-c

12			
0.44 eV			
O	-0.57158568	5.89975424	0.62996597
O	-1.28686722	3.48630857	1.00859467
O	0.17136523	4.54609965	-1.37411093
Si	-0.56438777	4.39537909	-0.01184288
Mg	-1.56985903	5.03373809	2.20546483
Mg	0.39529724	6.44740664	-1.01508208
O	-3.35664345	5.83708919	2.72297956
O	-3.62720555	6.28208493	3.83325159
O	-0.76711581	5.27044697	4.0503336
O	-1.34385337	5.77993588	5.00514957
O	1.12047614	8.13590105	-1.72417823
O	1.07492925	9.2364357	-1.16398566

(2,1,9)-d

12			
0.53 eV			
O	-1.3666696	5.52063565	0.68656727
O	-1.82049919	3.06937762	0.45915748
O	0.86993539	3.86505334	0.2853398
Si	-0.63427319	4.12803544	0.45991802
Mg	-2.98843272	4.53843929	0.72487403
Mg	2.59352917	3.4068972	-0.23133565
O	3.16340288	2.52422408	1.85089539
O	4.05296115	1.83279386	2.23959231
O	3.45237865	3.21216154	-1.95750943
O	3.87198243	2.26575837	-1.11736849
O	3.84018256	5.20637377	0.37087969
O	4.81547245	5.70739984	-0.09423044

[1] Bakker, J. M.; Lapoutre, V. J. F.; Redlich, B.; Oomens, J.; Sartakov, B. G.; Fielicke, A.; von Helden, G.; Meijer, G.; van der Meer, A. F. G., Intensity-Resolved Ir Multiple Photon Ionization and Fragmentation of C60. J. Chem. Phys. 2010, 132, 074305.
[2] Haertelt, M.; Lapoutre, V. J. F.; Bakker, J. M.; Redlich, B.; Harding, D. J.; Fielicke, A.; Meijer, G., Structure Determination of Anionic Metal Clusters Via Infrared Resonance Enhanced Multiple Photon Electron Detachment Spectroscopy. J. Phys. Chem. Lett. 2011, 2, 1720-1724.
[3] Meng Wang, G. Audi, F.G. Kondev, W.J. Huang, S. Naimi, Xing Xu, Chinese Physics C Vol. 41, No. 3 (2017) 030003
[4] Meng Wang, G. Audi, F.G. Kondev, W.J. Huang, S. Naimi, Xing Xu, Chinese Physics C Vol. 41, No. 3 (2017) 030003
[5] D. J. Wales, J. P. K. Doye, Global Optimization by Basin-Hopping and the Lowest Energy Structures of Lennard-Jones Clusters Containing up to 110 Atoms. *J. Phys. Chem. A*. 1997, 101, 5111–5116.

[6] A. Macià Escatllar, T. Lazaukas, S. M. Woodley, S. T. Bromley, Structure and Properties of Nanosilicates with Olivine (Mg_2SiO_4) N and Pyroxene ($MgSiO_3$) N Compositions. *ACS Earth Sp. Chem*. 2019, 3, 2390–2403.
[7] C. Adamo, V. Barone, Toward reliable density functional methods without adjustable parameters: The PBE0 model. *J. Chem. Phys*. **1999**, 110, 6158−6170.
[8] Jensen, S. R.; Saha, S.; Flores-Livas, J. A.; Huhn, W.; Blum, V.; Goedecker, S.; Frediani, L. The Elephant in the Room of Density Functional Theory Calculations. *J. Phys. Chem. Lett*. **2017**, 8, 1449− 1457.
[9] A. Tkatchenko, M. Scheffler, Accurate Molecular Van Der Waals Interactions from Ground-State Electron Density and Free-Atom Reference Data, *Phys. Rev. Lett.* 102, 073005.
[10] Blum, V.; Gehrke, R.; Hanke, F.; Havu, P.; Havu, V.; Ren, X.; Reuter, K.; Scheffler, M. Ab initio molecular simulations with numeric atom-centered orbitals. *Comput. Phys. Commun*. **2009**, 180, 2175− 2196.
[11] S. Bhattacharya, S. V. Levchenko, L. M. Ghiringhelli, M. Scheffler, Stability and Metastability of Clusters in a Reactive Atmosphere: Theoretical Evidence for Unexpected Stoichiometries of Mg_MO_x, *Phys. Rev. Lett.* 2013, 111, 135501.
[12] B. E. Krisyuka, A. V. Maiorovb, Quantum Chemical Study of the Primary Step of Ozone Addition at the Double Bond of Ethylene, *Kinet. Catal.*, 2011, 52, 798.
[13] Valencia, E. M.; Worth, C. J.; Fortenberry, R. C. Enstatite ($MgSiO_3$) and forsterite (Mg_2SiO_4) monomers and dimers: highly detectable infrared and radioastronomical molecular building blocks. Mon. Not. R. Astron. Soc. 2020, 492, 276–282.
[14] de la Pierre, M.; Orlando, R.; Maschio, L.; Doll, K.; Ugliengo, P.; Dovesi, R. Performance of Six Functionals (LDA, PBE, PBESOL, B3LYP, PBE0, and WC1LYP) in the Simulation of Vibrational and Dielectric Properties of Crystalline Compounds. The Case of Forsterite Mg_2SiO_4. *J. Comput. Chem*. **2011**, 32, 1775−1784.
[15] Demichelis, R.; Suto, H.; Nöel, Y.; Sogawa, H.; Naoi, T.; Koike, C.; Chihara, H.; Shimobayashi, N.; Ferrabone, M.; Dovesi, R. The infrared spectrum of ortho-enstatite from reflectance experiments and first-principle simulations. *Mon. Not. R. Astron. Soc*. **2012**, 420, 147− 154.
[16] Mariñoso Guiu, J.; Macia Escatllar, A.; Bromley, S. T. How Does Temperature Affect the Infrared Vibrational Spectra of Nanosized Silicate Dust? *ACS Earth Space Chem*. **2021**, 5, 4, 812–823.
[17] J. P. Perdew, K. Burke, and M. Ernzerhof, Generalized gradient approximation made simple, *Phys. Rev. Lett.*, **1996**, 77, 3865-3868.
[18] K. P. Huber, G. Herzberg, Molecular Spectra and Molecular Structure Iv. Constants of Diatomic Molecules; Van Nostrand, New York, 1979
